# Effects of preoperative oral enzyme-hydrolyzed rice flour solution on gastric emptying and insulin resistance in patients undergoing laparoscopic cholecystectomy: a prospective randomized controlled trial

**DOI:** 10.1186/s12871-023-02012-4

**Published:** 2023-02-13

**Authors:** Yang Yuan, Guangjun Shi, Huailong Chen, Mingshan Wang, Haofei Liu, Xiao Zhang, Bin Wang, Gaofeng Zhang, Lixin Sun

**Affiliations:** 1 Department of Anesthesiology, Qingdao Municipal Hospital, University of Health and Rehabilitation Sciences, Qingdao, 266071 Shandong People’s Republic of China; 2 Department of Hepatobiliary Pancreatic Surgery, Qingdao Municipal Hospital, University of Health and Rehabilitation Sciences, Qingdao, 266071 Shandong People’s Republic of China; 3 Department of Anesthesiology, Qingdao Eight People’s Hospital, Qingdao, 266041 Shandong People’s Republic of China; 4 Graduate School, Qingdao University, Qingdao, 266071 Shandong People’s Republic of China

**Keywords:** Enhanced recovery after surgery, Perioperative oral carbohydrate, Laparoscopic cholecystectomy, Insulin resistance, Antrum ultrasonography

## Abstract

**Background:**

The effect of preoperative oral carbohydrates (POC) on insulin resistance (IR) of laparoscopic cholecystectomy (LC) remains debatable. Enzyme-hydrolyzed rice flour (EHR) is a kind of water-soluble micromolecular carbohydrates. This study aimed to investigate the impact of preoperative oral EHR solution on gastric emptying and IR in patients undergoing LC.

**Methods:**

Patients (*n* = 100) undergoing LC were divided into oral-water group (group C) or oral-EHR solution (group E) randomly (*n* = 50 each), and the patients drank 300 ml water or EHR solution 2-3 h before surgery respectively. Gastric emptying which was quantized by gastric volume (GV) from antrum ultrasonography, IR indicators, subjective comfort indicators, handgrip strength, postoperative recovery indexes, and complications were recorded.

**Results:**

There were no differences in GV between the two groups before oral administration (V_0_), immediately after oral administration (V_1_) and before anesthesia induction(V_2_). The GV at V_2_ (GV_2_) reduced to the level of V_0_ (GV_0_) in the two groups. Fasting glucose (FG), fasting insulin (FINS) and Homa-IR in the two groups increased at postoperative day 1 (Pos 1d) compared with those at preoperative day 1(Pre 1d). Homa-IS and Homa-β in the two groups decreased at Pos 1d compared with those at Pre 1d. FG, FINS and Homa-IR in group E were lower than those in group C at Pos 1d, and Homa-IS and Homa-β were higher in group E than those in group C at Pos 1d. Subjective comfort indictors (hunger, fatigue and anxiety) in group E were lower than those in group C at preoperative 15 min (Pre 15 min) and postoperative 1 h (Pos 1 h). Handgrip strength in group E was raised compared with that in group C at Pre 15 min, Pos 1 h and Pos 1d. There was a lower incidence of nausea and earlier exhaust time in group E.

**Conclusion:**

Oral 300 ml EHR solution 2-3 h before LC surgery did not increase the occurrence of reflux and aspiration during anesthesia induction with a normal gastric emptying, ameliorated postoperative IR, improved subjective comfort, and promoted postoperative gastrointestinal function recovery.

**Trial registration:**

Prospectively registered at the China Clinical Trial Registry, registration number: ChiCTR2000039939, date of registration:14/11/2020.

## Introduction

Surgical trauma and anesthesia factors can inevitably induce postoperative insulin resistance (IR) [1]. Traditional strategy of preoperative starvation aiming to prevent regurgitation and aspiration of stomach contents exacerbated the level of postoperative IR [2]. IR was a state of glycolipid metabolism disorder which increased endogenous glucose production, lessened glycogen synthesis and gluconeogenesis in hepatocytes, inhibited lipolysis metabolism, and finally lead to excessive consumption of triglyceride (TG) and protein storage [3]. Research showed that IR contributed to postoperative infection and other complications, prolonged hospital stays [4].

Preoperative oral carbohydrates (POC) treatment at least 2–3 h before surgery, as one of the important parts of enhanced recovery after surgery (ERAS), has been proved to bring perioperative benefits and promote recovery for patients [5]. Nowadays, there are multiple kinds of carbohydrates which develop inconveniences for homogeneous management and propaganda of POC among different departments. And some kinds are expensive to increase patient hospital expenses. Enzyme-hydrolyzed rice flour (EHR) is a cheap production containing various micromolecular saccharides which is converted from macromolecular starch through enzymolysis technology. EHR is easily soluble in water, and the solution is a clear liquid which meets the carbohydrate standard in ERAS guidelines [6]. Laparoscopic cholecystectomy (LC) is an effective treatment for gallbladder stones and cholecystitis. Although LC is a minimally invasive surgery with less trauma, it is also accompanied with stress response and postoperative IR [7]. POC has been proved to reduce IR in most types of surgery [8, 9], however the effect of POC on LC remains controversial [10, 11].

Previous research showed that antrum ultrasonography can accurately measure the quality and quantity of gastric contents [12]. In our preliminary study for 30 healthy volunteers, the outcomes showed that the gastric content measured by antrum ultrasonography returned to the fasting level 2 h after oral EHR solution. Therefore, we conducted a randomized prospective study to evaluate the gastric emptying by bedside antrum ultrasonography and the influence on IR of oral EHR solution 2-3 h before LC operation.

## Methods

### Study design and participants

Gastric emptying and insulin resistance index were two primary outcomes in this study. We assumed the sample size based on the two outcomes respectively through setting the parameters of 90% power, 5% of a type 1 error, 10% of missing rate, and equivalent cases. In our pre-experiment, the mean of gastric volume (GV) 2 h after oral liquid in both groups are 38 ml, the combined standard deviation of the two groups was 10 ml, and the non-inferiority margin was -6 ml. Each group would need 48 patients using non-inferiority tests for two means in term of gastric emptying. According to previous study [13] and our pre-experiment results, the insulin resistance (IR) index in group C was 3.5 ± 0.9, while it was 2.9 ± 0.8 in group E at postoperative day 1 (Pos 1d). Thus 50 patients in each group would be required. Finally, 100 of 110 patients were recruited in the study.

This prospective, randomized and controlled study was approved from Ethics Committees of the Qingdao Municipal Hospital (XCJJ No. 014 (fast) in 2020), and registered in the center of Chinese Clinical Trial Registry (ChiCTR2000039939). Written informed consent was obtained from participating hospitalization patients. A total of 110 patients between January 2021 and August 2021 in the hepatobiliary surgery of Qingdao Municipal Hospital was enrolled in the study who were divided into two groups: oral-water group (group C) and oral-EHR solution group (group E). The inclusion criteria were as follows: patients undergoing elective laparoscopic cholecystectomy before 12 am; 18–64 years old; American society of anesthesiologists (ASA) classification of grade I or II and cardiac function of grade I or II; body mass index (BMI) between 19–30 kg/m^2^. The exclusion criteria included patients with gastric emptying disorders (gastroesophageal reflux and digestive tract obstruction, etc.); with diabetes mellitus; with a history of abdominal operation; with a history of alcohol dependence or drug abuse; allergic to maltodextrin; women in pregnancy or lactation period; patients who cannot understand contents of VAS scores due to communication or cognition disorders.

### Randomization and masking

The patients were assigned to group C or group E randomly and equally using a random number table method. All patients started fasting after supper and were forbidden from fluid intake after 24:00 the day before surgery. Patients in group C were required to drink 300 ml clear water, while patients in group E needed to ingest 300 ml solution of 2.5 packets of EHR (20 g/packet, Bangshidi (Guangdong) Medical Food Co., Ltd) within 5 min that were provided by nurses in ward 2–3 h prior to surgery. According to the instructions of EHR production, it contains 93 g of carbohydrate, 2 g of dietary fiber and 200 mg of sodium, and no protein or fat per 100 g of EHR. The patients didn’t know which kinds of liquids they drunk. The anesthesiologists who implemented anesthesia or evaluated the following indicators (antrum ultrasonography, insulin resistance indicators, handgrip strength, complications) were blinded to the assignment.

### Anesthesia

Procedures of anesthesia and operation were carried out by one fixed group of anesthesiologists and surgeons. After NBP, ECG, SpO_2_, temperature and BIS index were monitored, sequential induction of general anesthesia was initiated with 0.05 mg·kg^−1^ midazolam, 0.3 mg·kg^−1^ etomidate, 0.3–0.5 μg·kg^−1^ sufentanil, and 0.10–0.15 mg·kg^−1^ cisatracurium intravenously. Then, volume controlled mechanical ventilation was applied after endotracheal intubation, and the ventilatory settings were adjusted to keep the PetCO_2_ at 35–50 mmHg. Anesthesia was maintained by inhalational 1.5%-3% sevoflurane, intermittent infusion of cisatracurium, continuous infusion of 0.2–0.5 μg·kg^−1^·min^−1^ remifentanil and 0.2–0.7 μg·kg^−1^·h^−1^ dexmedetomidine. The NBP and HR fluctuated ranging ± 20 of baseline, and the BIS was maintained between 40–60 through adjusting the dosage of sedation analgesia and vasoactive medicines. Before skin suture, incision infiltration with 0.375% ropivacaine was performed for postoperative analgesia.

### Assessment of gastric emptying

One fixed anesthesiologist who has received professional antrum ultrasonic training performed the bedside antrum ultrasonic scanning before oral liquid (V_0_), immediately after oral liquid (V_1_) and before induction (V_2_) to assess the gastric emptying. According to a previously depicted scanning protocol [14], the patient was laid in the right lateral decubitus position, and a low frequency (2 to 5 MHz), curvilinear array transducer (M-Turbo, Sonosite Co., Ltd, American) was slidden from the midsagittal to right parasagittal plane to identify the gastric antrum between the left lobe of the liver and the pancreas, at the level of the aorta or inferior vena cava. The image of gastric antrum was frozen at rest rather than during peristaltic contractions, and the anteroposterior (AP) and craniocaudal (CC) antral diameters were measured. The cross-sectional area of the antrum (CSA) was calculated using the mathematical model CSA = π × (AP × CC ÷ 4). The mean of three measurement was used to calculate the gastric volume (GV) on the basis of Perlas model (2013) formula [15]: GV(ml) = 27.0 + 14.6 × CSA(cm^2^)-1.28 × age. Then the ΔGV (GV1-GV0) was obtained.

### Insulin resistance indicators

Antecubital venous blood samples were collected at preoperative day 1 (Pre 1d), and postoperative day 1(Pos 1d) to test the serum levels of fasting glucose (FG) and insulin (FINS) via an chemistry automatic analyzer and radioimmunoassay method respectively. The homeostatic model assessment (Homa) was most commonly used to evaluate the fasting related indicators of insulin resistance in clinical practice [16]. The insulin resistance index was calculated as Homa-IR = FG × FINS /22.5, the insulin secretion index was calculated as Homa-β = 20 × FINS/(FG-3.5) × 100%, and the insulin sensitivity index was calculated as Homa-IS = 1/Homa-IR. The unit of glucose was mmol·L^−1^, and the insulin was m IU·L^−1^ in above formulas. We assessed the above indicators at Pre 1d and Pos 1d.

### Subjective comfort indicators, handgrip strength and other indicators

Visual analog scale (VAS) score was used to evaluate the subjective comfort indicators (thirst, fatigue, hunger and anxiety) at Pre 1d, Pre 15 min and Pos 1 h. The scores were composed of ten vertical lines from the left to right, the left-most and right-most vertical line indicated “not undergoing the discomfortable symptom” and “the worst emotional experience” that corresponded to the score from 0 to 10. The handgrip strength of dominant hand was measured using a corrected grip dynamometer (CAMRY EH101, SENSSUN Co. Ltd, Guangdong, China) at Pre 1d, Pre 15 min, Pos 1 h and Pos 1d. Adjusted distance of the dynamometer to the second joint of the index finger in accordance with the size of dominant hand to ensure the measurement precision. According to previous study [17], the patients lied in the + 30° semi-recumbent position: shoulders adducted and neutrally rotated, elbow flexed, upper limb leaned on bed, wrist neutrally positioned. The patients completed 3 consecutive maximal isometric contraction for 3 s with10-30 s interval. The mean of 3 measurements was recorded as the handgrip strength.

The occurrence of gastroesophageal reflux and aspiration during anesthesia induction, adverse reaction including nausea, vomiting within 24 h postoperative, the exhaust time, postoperative complications, reoperation rate, infection and the hospital stay were recorded.

### Statistical analysis

SPSS 22.0 software (SPSS, Inc., Chicago, IL) was used for all statistical analysis. The measurement data in line with normal distribution were presented as the means ± standard deviation ($\overline{x}$ ± *s*). Independent samples *t* test was used for comparison between two groups. Single-sample *t* test was used for comparison between the sample mean and population mean. Paired* t* test was used for comparison between two different time points in the same group. Repeated measures analysis of variance (ANOVA) was used to determine the difference among time points in the same group, followed by least significant difference post hoc test. The measurement data of abnormal distribution were presented as median (interquartile range (IQR)), and were analyzed by Mann–Whitney *U* test. The difference of enumeration data was detected by *χ* ^2^ test or Fisher's exact test. A value of* P* < 0.05 was recognized as statistically significant.

## Results

**Fig. 1:**
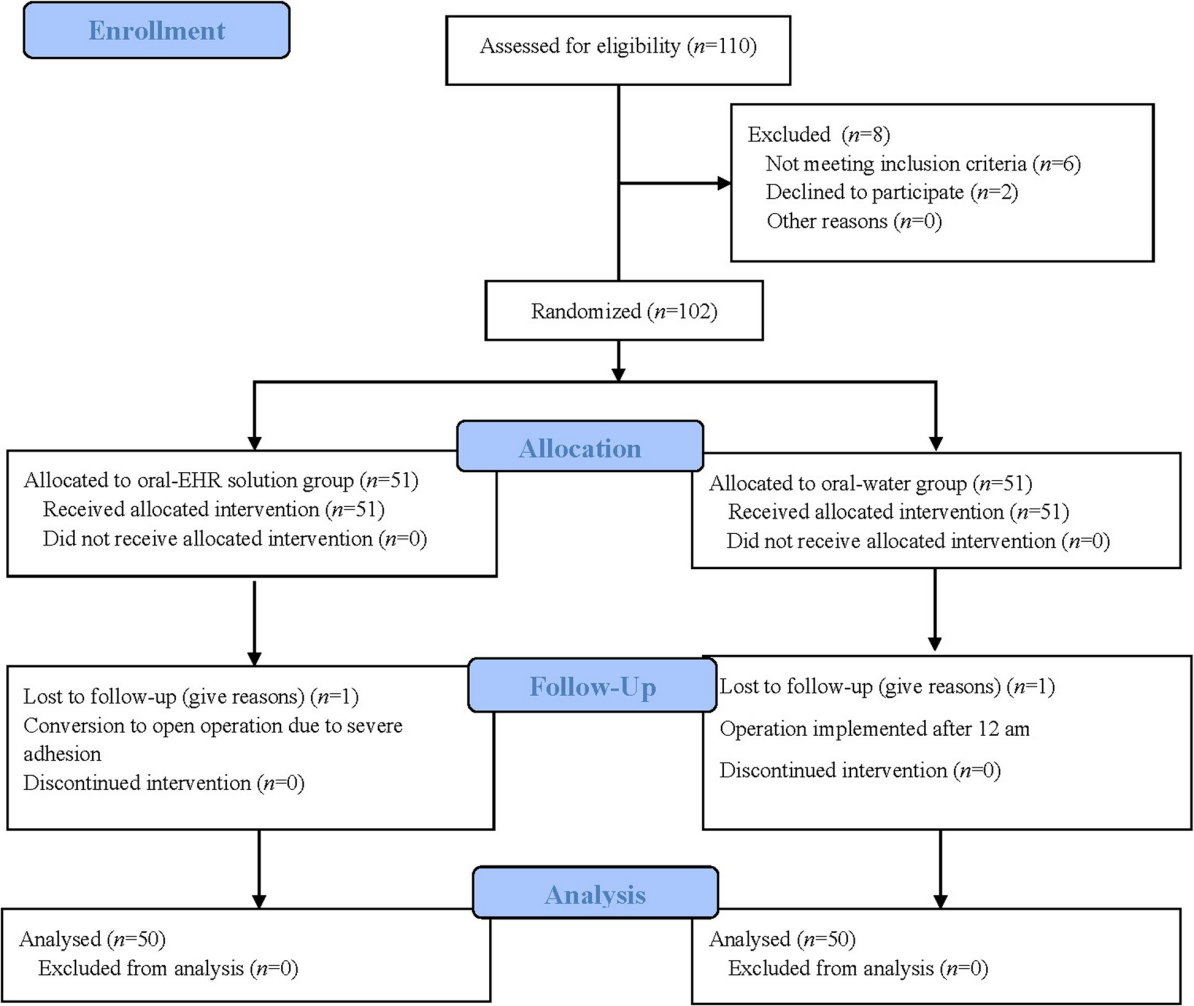
Flow-chart of patient selection

In total, 110 patients who underwent LC between January 2021 and March 2021 were enrolled in this study. Ultimately, there were 50 patients who completed the study in each group. The study scheme is shown in Fig. 1.

Univariable analysis shows that demographics including gender ratio, age, BMI and ASA classification were similar between the two groups (*P* > 0.05). In general, there were no significant differences in time between intake and induction, operative duration and the intraoperative blood loss (*P* > 0.05, Table 1).

**Table 1:** Baseline characteristics of the two groups

	Group C (*n* = 50)	Group E (*n* = 50)	*P*-value
Gender (*n*, %)			
Male	28(56)	26(52)	
Female	22(44)	24(48)	0.668
Age (year, mean ± SD)	52.3 ± 9.2	53.1 ± 8.2	0.626
BMI (kg/m^2^, mean ± SD)	25.3 ± 3.9	25.2 ± 4.0	0.910
ASA classification (*n*, %)			
I	24(48)	22(44)	
II	26(52)	28(56)	0.668
Time between intake and induction (min, mean ± SD)	147.3 ± 25.1	149.1 ± 24.2	0.716
Operative duration (min, mean ± SD)	65.6 ± 19.4	66.8 ± 19.0	0.747
blood loss (ml, M (P25, P75))	15(10,20)	15(10,15)	0.733

*BMI* Body mass index, *ASA* American Society of Anesthesiologists, *SD* Standard deviation, *M* Median, *P* Percentile

*P*-value indicates the statistical difference between the two groups

### Gastric volume under gastric ultrasound

We rechecked the applicability of Perlas model (2013) formula for the two kinds of drinks in the study population. The single-sample *t* test showed that ΔGV (GV_1_-GV_0_) in each group and the total ΔGV was similar to actual drinking volume 300 ml (*P* > 0.05, Table 2) which indicated the accuracy of the formula with regards to water or EHR solution.

**Table 2:** ΔGV and actual drinking volume in the two groups

	ΔGV (ml, mean ± SD)	actual drinking volume(ml)	*P*-value
Group C (*n* = 50)	298.2 ± 11.0	300	0.260
Group E (*n* = 50)	300.2 ± 10.6	300	0.887
Total (*n* = 100)	299.2 ± 10.9	300	0.470

*GV*_*0*_ Gastric volume before oral liquid, *GV*_*1*_ Gastric volume immediately after oral liquid, *GV*_*2*_ Gastric volume before induction, ΔGV = GV_1_-GV_0_, *SD* Standard deviation

*P*-value indicates the statistical difference between the ΔGV and actual drinking volume

**Fig. 2:**
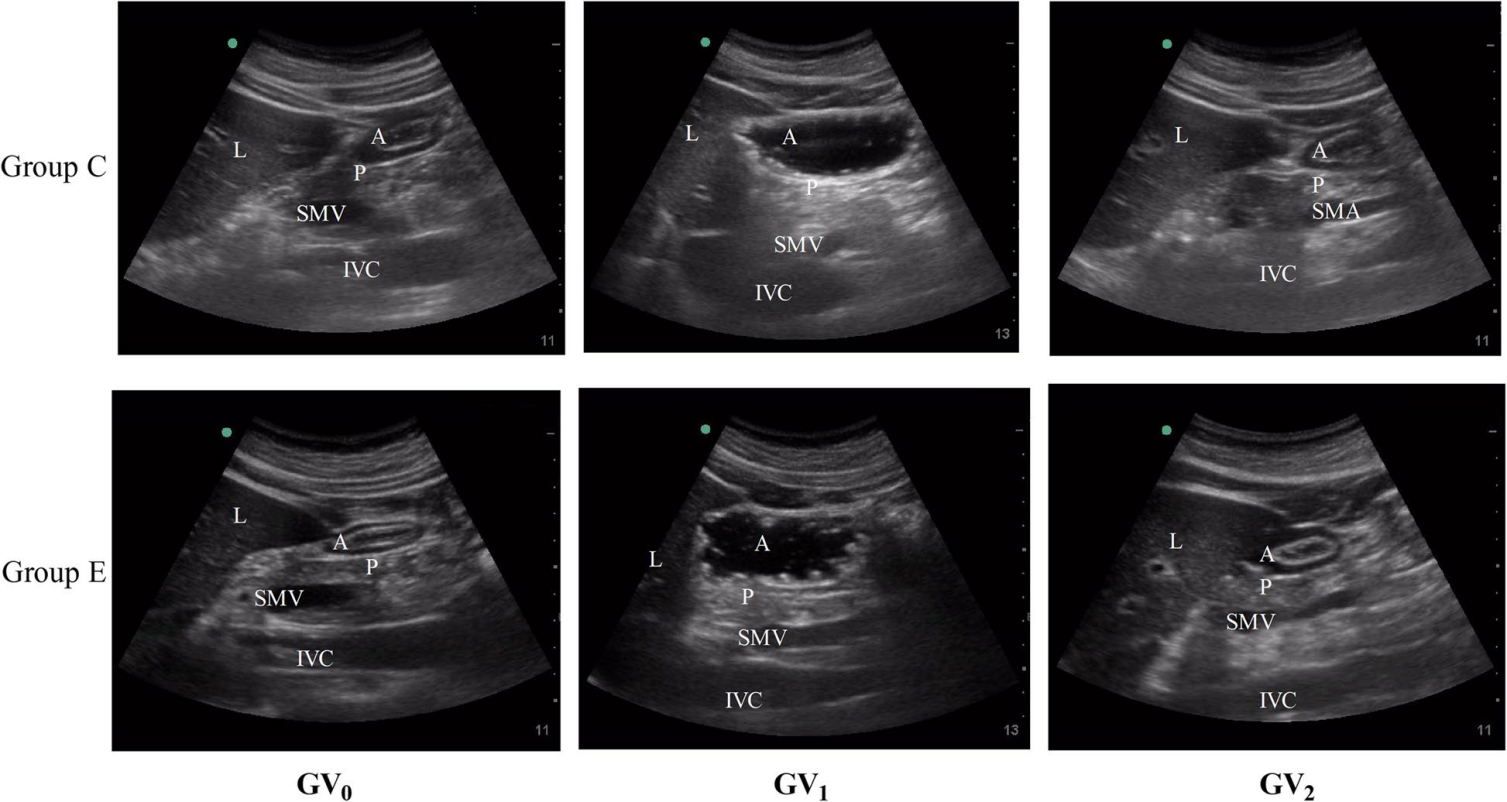
Sagittal scan of the antrum at different time points in the two groups. A = gastric antrum, L = liver, P = pancreas, SMA = superior mesenteric artery, IVC = inferior vena cava, SMV = superior mesenteric vein, Group C = oral-water group, Group E = oral- EHR solution group, GV0 = gastric volume before oral administration, GV1 = gastric volume immediately after oral administration, GV2 = gastric volume before anesthesia induction

We detected the gastric emptying of the two kinds of drinks using the ultrasound and the formula (Fig. 2). There were no significant differences between the two groups with respect to GV_0_, GV_1_ and GV_2_ (*P* > 0.05). The GV_1_ had a significant increase in comparison with GV_0_ (*P* < 0.01), however the GV_2_ shrank to the level of GV_0_ in both of the groups (*P* > 0.05, Table 3).

**Table 3:** Gastric volume under gastric ultrasound in the two groups

	Group C (*n* = 50)	Group E (*n* = 50)	*P*-value
GV_0_ (ml, mean ± SD)	37.0 ± 7.9	38.4 ± 7.0	0.352
GV_1_ (ml, mean ± SD)	335.2 ± 14.1^*^	338.6 ± 14.3^*^	0.237
GV_2_ (ml, mean ± SD)	38.4 ± 6.9	37.1 ± 8.3	0.394

GV_0_, gastric volume before oral liquid; GV_1_, gastric volume immediately after oral liquid; GV_2_, gastric volume before induction; SD, standard deviation

*P*-value indicates the statistical difference between the two groups at the same time point

^*^Indicates a statistically significant difference compared with GV_0_, *P* < 0.01

### Insulin resistance indicators

The levels of FG, FINS and Homa-IR increased, and the levels of Homa-IS and Homa-β declined at Pos 1d compared with those at Pre 1d in the two groups respectively (*P* < 0.05).

There was no difference in the baseline of blood glucose, insulin, Homa-IR, Homa-IS, and Homa-β at Pre 1d between the two groups (*P* > 0.05). However, the values of FG, FINS and Homa-IR in group E were lower than those in group C (*P* < 0.05), while the values of Homa-IS and Homa-β in group E were higher than those in group C (*P* < 0.05) at Pos 1d. (Table 4).

**Table 4:** Insulin resistance indicators in the two groups

	Group C (*n* = 50)	Group E (*n* = 50)	*P*-value
FG(mmol/L, mean ± SD)			
Pre 1d	5.2 ± 0.9	5.2 ± 0.7	0.666
Pos 1d	7.4 ± 1.2^*^	6.7 ± 1.2^*^	0.010
FINS(mU/L, mean ± SD)			
Pre 1d	7.6 ± 1.4	7.5 ± 1.7	0.706
Pos 1d	10.8 ± 1.9^*^	9.6 ± 2.2^*^	0.005
Homa-IR (mean ± SD)			
Pre 1d	1.8 ± 0.6^a^	1.8 ± 0.6^a^	0.609
Pos 1d	3.6 ± 1.2^*^	3.0 ± 1.2^*^	0.007
Homa-IS (M(P25, P75))			
Pre 1d	0.5(0.4,0.8)^a^	0.6(0.5,0.8)^a^	0.605
Pos 1d	0.3(0.2,0.4)^*^	0.4(0.3,0.5)^*^	0.018
Homa-β (M(P25, P75))			
Pre 1d	81.0(67.2,173.2)^a^	89.5(75.9,117.3)^a^	0.504
Pos 1d	58.4(50.0,61.1)^*^	60.9(53.4,66.8)^*^	0.045

*FG* Fasting glucose, *FIns* Fasting insulin, *HOMA-IR* Homeostasis model assessment-insulin resistance index, *HOMA-IS* Homeostasis model assessment-insulin sensitivity index, *HOMA-β* Homeostasis model assessment-β; *Pre 1d* Preoperative day 1, *Pos 1d* Postoperative day 1, *SD* Standard deviation, *M* Median, *P* Percentile

*P*-value indicates the statistical difference between the two groups at the same time point

^*^Indicates a statistically significant difference compared with Pre 1d, *P* < 0.05

^a^Indicates values that were not subject to normal distribution (ManneWhitney *U* test)

### Subjective comfort parameters and handgrip strength

We compared the VAS scores of subjective comfort parameters (thirst, fatigue, hunger and anxiety) and handgrip strength between the two groups. All the above indicators between the two group were at the similar levels at Pre 1d (*P* > 0.05). There was no distinct difference in the VAS score of thirst between the two group at Pre 15 min and Pos 1 h (*P* > 0.05). The VAS scores of fatigue, hunger and anxiety in group E were significantly lower than those in group C both at Pre 15 min and Pos 1 h (*P* < 0.05 or 0.01). The handgrip strength in group E was higher than that in group C at Pre 15 min, Pos 1 h and Pos 1d (*P* < 0.05, Table 5).

**Table 5:** Subjective comfort parameters and handgrip strength in the two groups

	Group C (*n* = 50)	Group E (*n* = 50)	*P*-value
Thirst (score, mean ± SD)			
Pre1d	1.5 ± 0.5	1.4 ± 0.6	0.858
Pre 15 min	1.2 ± 0.4^*^	1.2 ± 0.4^*^	0.806
Pos 1 h	3.1 ± 0.8^*^	2.9 ± 0.8^*^	0.132
Fatigue (score, mean ± SD)			
Pre1d	3.0 ± 0.8	3.0 ± 0.7	0.796
Pre 15 min	3.1 ± 0.8^*^	2.7 ± 0.7^*^	0.010
Pos 1 h	3.9 ± 1.2^*^	3.3 ± 1.1^*^	0.007
Hunger (score, mean ± SD)			
Pre1d	3.1 ± 0.9	3.0 ± 0.7	0.616
Pre 15 min	3.2 ± 1.0	2.6 ± 0.8^*^	0.002
Pos 1 h	4.0 ± 1.0^*^	3.3 ± 1.1	0.001
Anxiety (score, mean ± SD)			
Pre1d	3.1 ± 0.9	3.0 ± 0.8	0.907
Pre 15 min	3.7 ± 0.9^*^	3.3 ± 0.9^*·^	0.025
Pos 1 h	3.5 ± 0.8^*^	3.1 ± 0.7	0.009
Handgrip strength (Kg, mean ± SD)			
Pre1d	31.2 ± 7.9	30.7 ± 7.4	0.750
Pre 15 min	31.4 ± 8.8	35.3 ± 9.9^*^	0.038
Pos 1 h	29.5 ± 8.0	33.6 ± 8.4	0.016
Pos 1d	30.8 ± 8.3	34.4 ± 8.4^*^	0.037

*Pre 1d* Preoperative day 1, *Pre 15 min* Preoperative 15 min, *Pos 1 h* Postoperative 1 h, *Pos 1d* Postoperative day 1, *SD* Standard deviation

*P*-value indicates the statistical difference between the two groups at the same time point

^*^Indicates a statistically significant difference compared with Pre 1d, *P* < 0.05

### Postoperative rehabilitation indicators and complications

The incidence of nausea in group E declined while compared with that in group C within 24 h after operation (*P* < 0.05). The time to first flatus in group E was earlier than that in group C (*P* < 0.05). There was no gastroesophageal reflux or aspiration happened during anesthesia induction in all the subjects. The hospital stay of the two groups was not different (Table 6). All the patients discharged uneventfully without complications including bile leaking, biliary injury, hemorrhage, incision infection or pneumonia. There were no reoperation or readmission occurred within 30 days after operation.

**Table 6:** Postoperative rehabilitation indicators and complications

	Group C (*n* = 50)	Group E (*n* = 50)	*P*-value
Nausea (*n*, %)	13(26)	5(10)	0.037
Vomiting (*n*, %)	3(6)	1(2)	0.617
first flatus (h, mean ± SD)	18.5 ± 5.0	16.2 ± 4.1	0.015
hospital stay (d, mean ± SD)	6.3 ± 1.4	6.2 ± 1.3	0.706

*SD* Standard deviation

*P*-value indicates the statistical difference between the two groups

## Discussion

Preoperative oral carbohydrate (POC) has been recognized as an important element of guidelines for ERAS, and received increasing attention from anesthesiologists [18, 19]. POC has been demonstrated to provided lots of benefits for patient, such as reducing traumatic inflammatory response [20], relieving postoperative insulin resistance (IR) and organ dysfunction, improving subjective comfort [21] and accelerating postoperative rehabilitation. However, the categories of carbohydrate drinks are abundant [22] which bring an extensive choice for preoperative carbohydrate loading, also cause problems for homogeneous management and preoperative publicizing and education across different departments of identical hospital. Moreover, the high price of most carbohydrate products increases hospital expenses. Therefore, it is important to provide clinical application with optimal carbohydrate drinks with characteristics of effortless gastric emptying, agreeable palatability, good patient compliance to implement, being economical, promoting postoperative physical status. Enzyme-hydrolyzed rice flour (EHR) solution is convenient for unified administration, propaganda education, large-scale promotion and clinical application. In the study, we evaluate the gastric emptying of oral EHR solution, and its effect on IR.

The published data [23] showed that in non-diabetes population the plasma insulin level reached the peak (> 10 times of fasting value) at about 1 h after intake of a mixed-meal containing 50 g of carbohydrates, and kept at 2–4 times of fasting value at 2-3 h after intake. Previous study recommended patients to ingest 300-400 ml solution containing 50 g of carbohydrates 2–3 h before surgery [19]. Thus, we dissolved 2.5 packages of EHR (50 g, containing 46.5 g carbohydrates) in 300 ml warm water for patients in group E. Scholars [13] compared the differences between preoperative oral single-dose carbohydrate and double-dose carbohydrates. The outcomes showed that POC at the night before surgery did not consolidate the influence of POC at 2-3 h before surgery on insulin resistance, subjective comfort, inflammation and immunity, instead disturb the patients’ sleep quality conversely. Accordingly, we chose the regime of single-dose EHR solution loading 2–3 h before surgery in the study.

Bedside antrum ultrasonography is an accurate, quick and effective technology to evaluate the properties and volume of gastric contents [24]. Meanwhile, the technology is easy to grasp. The success rate of anesthesiologists in evaluating the properties of gastric contents by ultrasound can reach 95% after proper training (about 33 repeated examination) [25]. The researchers in the study have grasped this ultrasonic scanning technique after being trained.

Anteroposterior (AP) and craniocaudal (CC) antral diameters in the right lateral decubitus (RLD) are measured to obtain the cross-sectional area of the antrum (CSA). Then the gastric volume can be calculated based on the formula of Perlas (2013) model [15] which is expressed as GV (ml) = 27.0 + 14.6 × CSA (cm^2^)-1.28 × age. The suitable range of the formula are 18–85 years, 45–110 kg and more than 145 cm heigh. Besides general population, further studies showed that the formula can be applied in obesity and pregnancy accurately [26, 27]. In the study, there is no difference between the ΔGV from the formula and the actual drinking volume 300 ml which further verify the applicability of the formula in estimating gastric emptying in the study.

The results of GV exhibited no significant differences between two groups at the three time points: before oral administration (V_0_), immediately after oral administration (V_1_) and before anesthesia induction(V_2_), and no differences between GV_2_ and GV_0_ in the two groups. The outcomes suggest that the gastric emptying rates of 300 ml water and EHR solution are similar, and both of them returned to the fasting value after 2–3 h. Meanwhile, there was no reflux or aspiration occurred during anesthesia induction, which indicated that 300 ml EHR solution loading 2–3 h before surgery cannot increase the risk of reflux aspiration.

Postoperative IR is a depressed state of glucose uptake and utilization by peripheral target organs and tissues, which result in abnormal metabolism of glucose, lipid and protein, and is associated with hyperglycemia reactions, infection and prolonged hospital stay [28]. The hyperinsulinemic euglycemic clamp (HEC) technique is the gold-standard to evaluate insulin sensitivity. However, the technique is hard to be widely applied in clinic because it is expensive, technically demanding and time-consuming. The homeostatic model assessment (Homa), which has been validated as a noteworthy correlation with HEC technique [29, 30], includes the indicators of Homa-IR, Homa-β, Homa-IS. It has been proposed that POC can reduce postoperative IR [31], we also observed the reduction of Homa-IR, and the increase of Homa-β, Homa-IS following preoperative oral EHR solution at the first day after operation.

There are various mechanisms behind the alleviation of postoperative IR induced by POC. Shi et al. [32] found that POC reduced postoperative IR via stimulating AMPK, which suppressed the phosphorylation of mTOR and insulin receptor substrate-1 (IRS-1) after colorectal resection. Also, study [33] indicated that POC attenuated the development of postoperative IR which was associated with the alternation pyruvate dehydrogenase kinase (PDK4) in muscle. The experiment in rats showed [34] that POC improves postoperative insulin sensitivity in skeletal muscles through the PI3K/AKT/mTOR pathway.

The main components of EHR are maltodextrin, oligosaccharide malt and polyglucose. Maltodextrin [35] is a kind of polysaccharide that is easily absorbable and relieves stress of digestive system for patients; oligosaccharide is characterized by low sweetness, low caloric, hypoosmolality, occurs continuous hydrolysis reaction in intestines which could increases blood glucose slightly and prolong the duration of energy supply; although polyglucose barely provide energy, as soluble cellulose, it increases the intestinal chyme volume and patients’ satiety, slows the absorption of carbohydrate, what is more, the short chain fatty acids produced from polyglucose by intestinal flora enhances intestinal mucosal barrier, reduce the absorption of endotoxin and play an important anti-inflammatory role which maybe one of the factors to relieve IR and promote intestinal peristalsis [36, 37]. As a result, in the study oral EHR solution decreased VAS scores of fatigue, hunger and anxiety, reduced the incidence of nausea, enhanced handgrip strength and shorten exhaust time. These are in accordance with the previous conclusions [38, 39] that POC improved the postoperative subjective well-being, reduced nausea and ameliorated gastrointestinal motility in surgical patients.

Skeletal muscle is one of the main target tissues of insulin. Insulin resistance disrupts both the amount and the timing of glucose into skeletal muscle [40]. Previous study [41] showed that POC inhibited the activity reduction of glycogen synthase in muscle biopsy, and maintain quadriceps femoris muscle strength while compared with placebo group. Gysel et al. [42] demonstrated that descending handgrip force were already present in healthy men with lower insulin sensitivity. Consequently, POC alleviated postoperative IR which would improve the uptake and storage of carbohydrate in skeletal muscle, and would be beneficial to ameliorate muscle function. Therefore, the decreased postoperative IR might be the reason for better handgrip strength in group E at Pos 1d in this study.

There are still limitations in the study. Firstly, a group that fasted before surgery, as a control to show the impact of preoperative fasting, was not set. Secondly, effects between glucose solution and enzyme-hydrolyzed rice flour solution were not compared. In addition, the similar research on other types of surgery needs to be further carried out.

## Conclusions

In conclusion, oral 300 ml of enzyme-hydrolyzed rice flour solution 2-3 h before surgery in patients undergoing laparoscopic cholecystectomy did not increase the occurrence of reflux and aspiration during anesthesia induction with a normal gastric emptying, ameliorated postoperative insulin resistance, improved the subjective comfort, and promoted postoperative gastrointestinal function recovery.

## Data Availability

The datasets used and/or analyzed during the current study are available from the corresponding author on reasonable request. Ethics approval and consent to participate. This study was approved by the Ethics Committees of the Qingdao Municipal Hospital(group) (XCJJ No. 014 (fast) in 2020), and registered in the center of Chinese Clinical Trial Registry (registration number: ChiCTR2000039939, date of registration:14/11/2020.). All methods were carried out in full accordance with the Declaration of Helsinki. Informed consent was obtained from all subjects and/or their legal guardian(s).

## Abbreviations

ASA: American Society of Anesthesiologists
AP: Anteroposterior
BMI: Body mass index
CC: Craniocaudal
CSA: Cross-sectional area of the antrum
EHR: Enzyme-hydrolyzed rice flour
ERAS: Enhanced Recovery After Surgery
FINS: Fasting insulin
FG: Fasting glucose
GV: Gastric volume
HEC: Hyperinsulinemic euglycemic clamp
HOMA: Homeostasis model assessment
HOMA-IR: Homeostasis model assessment-insulin resistance index
HOMA-IS: Homeostasis model assessment-insulin sensitivity index
HOMA-β: Homeostasis model assessment-β
IQR: Interquartile range
IR: Insulin resistance
LC: Laparoscopic cholecystectomy
PDK4: Pyruvate dehydrogenase kinase
POC: Preoperative oral carbohydrates
Pos 1d: Postoperative day 1
Pos 1 h: Postoperative 1 h
Pre 1d: Preoperative day 1
Pre 15 min: Preoperative 15 min
VAS: Visual analogue scales

## References

[1] Bagry HS, Raghavendran S, Carli F (2008). Metabolic syndrome and insulin resistance: perioperative considerations. Anesthesiology.

[2] Pimenta GP, de Aguilar-Nascimento JE (2014). Prolonged preoperative fasting in elective surgical patients: why should we reduce it?. Nutr Clin Pract.

[3] Lee SH, Park SY, Choi CS (2022). Insulin Resistance: From Mechanisms to Therapeutic Strategies. Diabetes Metab J.

[4] Blixt C, Ahlstedt C, Ljungqvist O, Isaksson B, Kalman S, Rooyackers O (2012). The effect of perioperative glucose control on postoperative insulin resistance. Clin Nutr.

[5] Ackerman RS, Tufts CW, DePinto DG, Chen J, Altshuler JR, Serdiuk A (2020). How Sweet Is This? A Review and Evaluation of Preoperative Carbohydrate Loading in the Enhanced Recovery After Surgery Model. Nutr Clin Pract.

[6] Nygren J, Thacker J, Carli F, Fearon KC, Norderval S, Lobo DN (2012). Guidelines for perioperative care in elective rectal/pelvic surgery: Enhanced Recovery After Surgery (ERAS(R)) Society recommendations. Clin Nutr.

[7] Micic D, Lalic N, Djukic V, Stankovic S, Trajkovic G, Oluic B (2018). Influence of IL-6, TNF-alpha and Hs-CRP on Insulin Sensitivity in Patients after Laparoscopic Cholecystectomy or Open Hernia Repair. J Med Biochem.

[8] Gustafsson UO, Scott MJ, Hubner M, Nygren J, Demartines N, Francis N (2019). Guidelines for Perioperative Care in Elective Colorectal Surgery: Enhanced Recovery After Surgery (ERAS((R))) Society Recommendations: 2018. World J Surg.

[9] Mortensen K, Nilsson M, Slim K, Schafer M, Mariette C, Braga M (2014). Consensus guidelines for enhanced recovery after gastrectomy: Enhanced Recovery After Surgery (ERAS(R)) Society recommendations. Br J Surg.

[10] Dock-Nascimento DB, de Aguilar-Nascimento JE, Magalhaes Faria MS, Caporossi C, Slhessarenko N, Waitzberg DL (2012). Evaluation of the effects of a preoperative 2-hour fast with maltodextrine and glutamine on insulin resistance, acute-phase response, nitrogen balance, and serum glutathione after laparoscopic cholecystectomy: a controlled randomized trial. JPEN J Parenter Enteral Nutr.

[11] Pedziwiatr M, Pisarska M, Matlok M, Major P, Kisielewski M, Wierdak M (2015). Randomized Clinical Trial To Compare The Effects Of Preoperative Oral Carbohydrate Loading Versus Placebo On Insulin Resistance And Cortisol Level After Laparoscopic Cholecystectomy. Pol Przegl Chir.

[12] Van de Putte P, Perlas A (2014). Ultrasound assessment of gastric content and volume. Br J Anaesth.

[13] Chen X, Li K, Yang K, Hu J, Yang J, Feng J (2021). Effects of preoperative oral single-dose and double-dose carbohydrates on insulin resistance in patients undergoing gastrectomy:a prospective randomized controlled trial. Clin Nutr.

[14] Perlas A, Davis L, Khan M, Mitsakakis N, Chan VW (2011). Gastric sonography in the fasted surgical patient: a prospective descriptive study. Anesth Analg.

[15] Perlas A, Mitsakakis N, Liu L, Cino M, Haldipur N, Davis L (2013). Validation of a mathematical model for ultrasound assessment of gastric volume by gastroscopic examination. Anesth Analg.

[16] Tepper S, Shahar DR, Geva D, Ish-Shalom S (2016). Differences in homeostatic model assessment (HOMA) values and insulin levels after vitamin D supplementation in healthy men: a double-blind randomized controlled trial. Diabetes Obes Metab.

[17] Flood A, Chung A, Parker H, Kearns V, O'Sullivan TA (2014). The use of hand grip strength as a predictor of nutrition status in hospital patients. Clin Nutr.

[18] Stumpo V, Staartjes VE, Quddusi A, Corniola MV, Tessitore E, Schroder ML (2021). Enhanced Recovery After Surgery strategies for elective craniotomy: a systematic review. J Neurosurg.

[19] Kotfis K,  Jamiol-Milc D, Skonieczna-Zydecka K, Folwarski M, Stachowska E (2020). The Effect of Preoperative Carbohydrate Loading on Clinical and Biochemical Outcomes after Cardiac Surgery: A Systematic Review and Meta-Analysis of Randomized Trials. Nutrients.

[20] Rizvanovic N, Nesek Adam V, Causevic S, Dervisevic S, Delibegovic S (2019). A randomised controlled study of preoperative oral carbohydrate loading versus fasting in patients undergoing colorectal surgery. Int J Colorectal Dis.

[21] Onalan E, Andsoy II, Ersoy OF (2019). The Effect of Preoperative Oral Carbohydrate Administration on Insulin Resistance and Comfort Level in Patients Undergoing Surgery. J Perianesth Nurs.

[22] Karimian N, Kaneva P, Donatelli F, Stein B, Liberman AS, Charlebois P (2020). Simple Versus Complex Preoperative Carbohydrate Drink to Preserve Perioperative Insulin Sensitivity in Laparoscopic Colectomy: A Randomized Controlled Trial. Ann Surg.

[23] Kudva YC, Carter RE, Cobelli C, Basu R, Basu A (2014). Closed-loop artificial pancreas systems: physiological input to enhance next-generation devices. Diabetes Care.

[24] Kruisselbrink R, Gharapetian A, Chaparro LE, Ami N, Richler D, Chan VWS (2019). Diagnostic Accuracy of Point-of-Care Gastric Ultrasound. Anesth Analg.

[25] Arzola C, Carvalho JC, Cubillos J, Ye XY, Perlas A (2013). Anesthesiologists' learning curves for bedside qualitative ultrasound assessment of gastric content: a cohort study. Can J Anaesth.

[26] Rouget C, Chassard D, Bonnard C, Pop M, Desgranges FP, Bouvet L (2016). Changes in qualitative and quantitative ultrasound assessment of the gastric antrum before and after elective caesarean section in term pregnant women: a prospective cohort study. Anaesthesia.

[27] Van de Putte P, Perlas A (2014). Gastric sonography in the severely obese surgical patient: a feasibility study. Anesth Analg.

[28] Tewari N, Awad S, Duska F, Williams JP, Bennett A, Macdonald IA (2019). Postoperative inflammation and insulin resistance in relation to body composition, adiposity and carbohydrate treatment: A randomised controlled study. Clin Nutr.

[29] Brennan AM, Standley RA, Yi F, Carnero EA, Sparks LM, Goodpaster BH (2020). Individual Response Variation in the Effects of Weight Loss and Exercise on Insulin Sensitivity and Cardiometabolic Risk in Older Adults. Front Endocrinol (Lausanne).

[30] Wallace TM, Levy JC, Matthews DR (2004). Use and abuse of HOMA modeling. Diabetes Care.

[31] Ljungqvist O, Scott M, Fearon KC (2017). Enhanced Recovery After Surgery: A Review. JAMA Surg.

[32] Shi M, Hu Z, Yang D, Cai Q, Zhu Z (2020). Preoperative Oral Carbohydrate Reduces Postoperative Insulin Resistance by Activating AMP-Activated Protein Kinase after Colorectal Surgery. Dig Surg.

[33] Awad S, Constantin-Teodosiu D, Constantin D, Rowlands BJ, Fearon KC, Macdonald IA (2010). Cellular mechanisms underlying the protective effects of preoperative feeding: a randomized study investigating muscle and liver glycogen content, mitochondrial function, gene and protein expression. Ann Surg.

[34] Wang Z, Liu Y, Li Q, Ruan C, Wu B, Wang Q (2015). Preoperative oral carbohydrate improved postoperative insulin resistance in rats through the PI3K/AKT/mTOR pathway. Med Sci Monit.

[35] Pogatschnik C, Steiger E (2015). Review of Preoperative Carbohydrate Loading. Nutr Clin Pract.

[36] Eslick S, Thompson C, Berthon B, Wood L (2022). Short-chain fatty acids as anti-inflammatory agents in overweight and obesity: a systematic review and meta-analysis. Nutr Rev.

[37] McNabney SM, Henagan TM (2017). Short Chain Fatty Acids in the Colon and Peripheral Tissues: A Focus on Butyrate, Colon Cancer, Obesity and Insulin Resistance. Nutrients.

[38] Cheng PL, Loh EW, Chen JT, Tam KW (2021). Effects of preoperative oral carbohydrate on postoperative discomfort in patients undergoing elective surgery: a meta-analysis of randomized controlled trials. Langenbecks Arch Surg.

[39] Makaryus R, Miller TE, Gan TJ (2018). Current concepts of fluid management in enhanced recovery pathways. Br J Anaesth.

[40] Merz KE, Thurmond DC (2020). Role of Skeletal Muscle in Insulin Resistance and Glucose Uptake. Compr Physiol.

[41] Henriksen MG, Hessov I, Dela F, Hansen HV, Haraldsted V, Rodt SA (2003). Effects of preoperative oral carbohydrates and peptides on postoperative endocrine response, mobilization, nutrition and muscle function in abdominal surgery. Acta Anaesthesiol Scand.

[42] Gysel T, Tonoli C, Pardaens S, Cambier D, Kaufman JM, Zmierczak HG (2016). Lower insulin sensitivity is related to lower relative muscle cross-sectional area, lower muscle density and lower handgrip force in young and middle aged non-diabetic men. J Musculoskelet Neuronal Interact.

